# Depression in adults with sickle cell disease: a systematic review of the methodological issues in assessing prevalence of depression

**DOI:** 10.1186/s40359-021-00543-4

**Published:** 2021-04-06

**Authors:** Damien Oudin Doglioni, Vincent Chabasseur, Frédéric Barbot, Frédéric Galactéros, Marie-Claire Gay

**Affiliations:** 1 EA4430 EvaCliPsy/ED139, Paris Nanterre University, Nanterre, France; 2 Red Blood Cell Genetic Diseases Unit (UMGGR), Teaching Hospital Henri Mondor, Créteil, France; 3 EA4430 EvaCliPsy, Paris Nanterre University, Nanterre, France; 4 INSERM Clinical Investigation Center 1429, Teaching Hospital Raymond Poincaré AP-HP, Garches, France; 5 French National Referral Centre for Sickle Cell Disease (MCGRE), Créteil, France

**Keywords:** Sickle cell disease, Depression, Prevalence, Methodology

## Abstract

**Background:**

Sickle cell disease (SCD) as other chronic medical conditions is commonly complicated by depression or other psychiatric symptoms. Results reported in studies present a large variation. Thus, synthetic data are needed to understand impact of depression in adults with SCD. The aim of this literature review is to analyse the methodology used in the studies assessing depression and discuss the different prevalence levels reported.

**Methods:**

Studies involving adults with SCD from 1999 to 2018 were included when providing data on prevalence of depression. It was defined by a psychometric assessment, a structured interview, or a medical record review. PRISMA recommendations were followed.

**Results:**

36 studies are included accordingly to our methodology. Prevalence variation is large, from 0% to more than 85%. We find that the type of assessment tool used plays a major role in this between studies variation. Also, methodological issues arise with respect to psychometric assessment. Moreover, differences emerge between continents, setting of recruitment or time of assessment.

**Conclusion:**

All these issues are discussed to provide insight on depression in adults with sickle cell disease.

**Trial Registration:**

*PROSPERO Registration* CRD42018100684.

## Background

Sickle cell disease (SCD) is the most common autosomal recessive disorder in humans [1], in which structurally abnormal haemoglobin leads to severe clinical manifestations such as haemolytic anaemia, greater susceptibility to infections and severe pain attacks [2]. People with SCD (pwSCD) are often affected by depression, and clinical evidence shows a link between emotional state and pathological events, particularly with regard to the major pain crises that remain its hallmark [3]. SCD is thus a major public health issue.

Depression is known to be the most common emotional disorder encountered in chronic diseases [4]. In 2017, the proportion of the world’s population with a depressive disorder was estimated at 3.59%, representing about 264 million people [5].

In international published studies, the prevalence of depressive disorders in medically ill patients is estimated to be between 12 and 61%, depending on the health condition [6]. For example, the worldwide prevalence is estimated between 12 and 40% in diabetes, 14.4% in asthma, 30% to 36% after a heart attack, 20% to 37% in patients with cancer, and 20% to 38% in coronary heart disease [7, 8].

The likelihood of having a comorbid depressive disorder with a chronic disease is significantly higher than having a depressive disorder alone [9]. Having a chronic disease significantly increases the risk of a depressive disorder by an odds ratio of 1.7–3.15 depending on the disease [10, 11].

Likewise, depressive disorders have a negative influence on the progression of chronic diseases. Patients with a chronic disease and a comorbid depressive disorder report significantly more medical symptoms (taking into account the severity of the disease) as compared to patients with chronic pathology alone [12]. In addition, such patients have significantly more hospitalisations for their disease compared to patients without a comorbid depressive disorder [11]. Considering SCD, more specifically, the literature indicates that patients with depression and SCD report increased hospitalizations and intensity of pain experienced as opposed to those with SCD alone [13–23]; regarding the frequency of pain attacks, studies indicate a higher frequency in patients where depression is also involved [17–19, 24, 25]. In particular, with regard to the interference of pain on the daily life of patients, studies show that in sickle cell disease depressed patients tend to feel a greater impact of pain on their lives than healthy patients [24, 26, 27]. These data are consistent with the literature in which the link between depression and pain has been explored (e.g. [28–30]).

It would be useful to have similar information in relation to pwSCD. Indeed, as for other chronic diseases, numerous studies have assessed depression in adults with SCD (awSCD), but the range of prevalence varies widely, depending on the assessment tool used: from 0% [31] to more than 85% [32]. The extent of the prevalence range reported emphasises the need for a review of the available data for researchers and clinicians. Accordingly, the aim of this literature review is to analyse the methodology used in the studies assessing depression and discuss the different prevalence levels reported.

## Methods

### Eligibility criteria

We reviewed studies involving awSCD from 1999 to 2018 included. Those involving patients with other psychiatric or medical comorbidities were considered only when the comorbidity was not an explicit inclusion criterion. We did not limit ourselves to patients with a diagnosis of a major depressive episode and considered studies assessing clinically significant depressive symptoms.

#### Types of outcomes

The evaluation of the prevalence of depression in awSCD was chosen as the primary outcome. It was defined by a psychometric assessment (quantitative data), a structured interview (qualitative data), or a medical record review (qualitative data).

#### Type of studies

We included research articles and reviews that provided relevant data. In particular, we have endeavoured to identify, in the methodology, the localisation and setting of recruitment, the characteristics of the patients, in particular the percentage of male patient, the average age and the genotype, and also the characteristics of the tools used, including cut-off in the case of psychometric tools.

Other kinds of publications were unsystematically screened to include relevant studies. In this analysis, only studies in English, French and Spanish languages were included.

### Search strategy

Eligible studies were identified from PubMed/Medline, ScienceDirect, PsychInfo/Article and a manual search within the references of the articles and reviews found. We only included published articles in peer-reviewed journal.

The search terms used were: “sickle cell” AND depression.

**Fig. 1:**
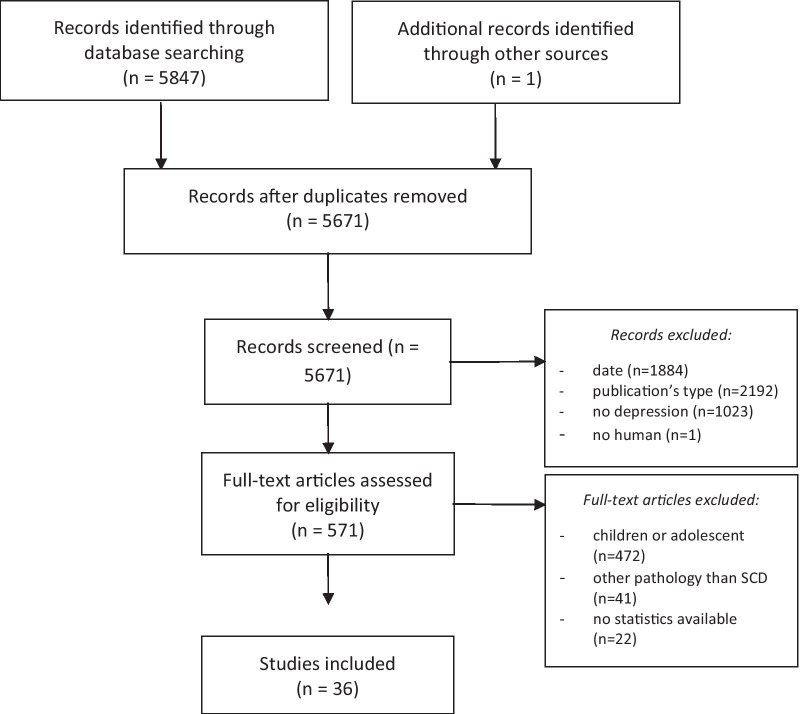
Flow chart (from: Moher et al. [34])

A comparison across the studies, samples and authors was carried out to avoid duplicates and a compilation of data from the same source. Figure 1 shows the steps taken in a flow chart.

### Data collection procedure

For this literature review, recommendations from the Preferred Reporting Items for Systematic Reviews and Meta-Analyses (PRISMA) were followed [33, 34]:

Step 1: selection of studies in accordance with our search terms, based on titles and abstract.
Step 2: in the selected studies at step 1, selection of studies respecting inclusion criteria, based on methodology:
  - only human involved,
  - only adults involved,
  - only sickle cell disease involved,
  - publication date from 1999 to 2018.
Step 3: in the selected studies at step 2, selection of studies with an evaluation of depression providing relevant and exploitable data.

A data extraction sheet based on the Cochrane Handbook for Systematic Reviews of Interventions’ guidelines (version 5.1.0, updated March 2011) was used [35] and adapted to our specific topic.

The protocol was registered in the PROSPERO database: CRD42018100684.

## Results

From 1999 to 2018, 36 cross-sectional published studies were found providing data on depression in pwSCD. These data are summarised in Table 1.

**Table 1:** Data summary

Year	Author	Methodology		Population			Measure			Results	
		Country	Recruitment setting	Sex (male, %)	Age (mean)	Genotype (SS, %)	Type	Name	Cut-off	N total	% depressed
1999	Wilson Schaeffer, J. J et al	USA	Outpatient	43.86	33.70		PA	CES-D 20	16	440	43.41
2000	Grant et al	USA	Outpatient	41.86	35.02^‡^		PA	CES-D 20	16	43	25.58
2003	Hasan et al	USA	Outpatient	54.00	36.00	94.00	PA	BDI-II	14	50	44.00
2005	Jenerette et al	USA	Cohort	28.00	35.00		PA	BDI 7	4	221	31.67
2006	Laurence et al	USA	Cohort	43.54		79.40	PA	CES-D 10	4	101	38.61
2007	Pells, J. et al	USA	Cohort		36.82		PA	LEMFPSCD (BDI-II)	14	67	35.82
2008	Levenson et al	USA	Cohort	38.40	34.00	70.96	PA	PHQ 9	10	232	27.59
2009	Carroll et al	USA	Inpatient	36.50^‡^	24.10^‡^		MRR		n/a	122	6.56
2009	Edwards et al	USA	Outpatient		36.82		PA	LEMFPSCD (BDI-II)	14	67	22.00
2010	Anie et al	Nigeria	Outpatient				PA	PISCD	n/a	253	44.27
2010	Asnani et al	Jamaica	Cohort	49.46	31.00		PA	BDI-II	14	277	21.66
2010	Kamble et al	USA	Outpatient	42.96	34.20		PA	BDI-II	14	142	35.21
2010	Mahdi et al	Bahrain	Outpatient	42.80	17.23^‡^	100	PA	DASS 21	10	243	59.26
2011	Carroll et al	USA	Inpatient				MRR		n/a	1874	4.27
2011	Treadwell et al	USA	Outpatient		31.20		PA	PHQ 9	10	77	61.04
2012	Rosine	Europe	Outpatient	44.30	35.60	100^†^	PA	BDI-II	14	89	49.44
2012	Vilela et al	Brazil	Outpatient	32.00	35.00	100	PA	BDI-II	20	24	16.67
2013	Gibson et al	Jamaica	Outpatient	48.00	36.40	70.00	PA	BDI-II	17	143	13.29
2014	Morgan et al	Jamaica	Outpatient		35.97	69.60	PA	BDI-II	14	140	15.00
2014	Wallen et al	USA	Cohort	49.00	34.00	85.00^†^	PA	BDI-II	16	315	20.63
2015	Mann-Jiles et al	USA	Outpatient	49.00		64.00	MRR		n/a	72	19.44
2015	Mastandréa et al	Brazil	Outpatient	38.20	30.00	72.70^†^	PA	PHQ 9	10	110	30.00
2015	Treadwell et al	USA	Cohort	40.00	31.60	73.00	PA	PHQ 9	10	77	32.47
2016	Al Sherawi et al	Oman	Outpatient Inpatient	34.00			PA	BDI-II	14	123	39.02
2016	Anim et al	Ghana	Outpatient	51.00		65.00	PA	BSI	?	201	0.00
2016	Fayand et al	Europe	Inpatient	43.00	27.00		MRR		n/a	439	5.24
2016	Ola et al	Nigeria	Outpatient	38.83	25.30	98.06	PA	PHQ 9	5	103	71.84
2016	Master et al	USA	Outpatient	48.00	33.00		PA	PROMIS	55	100	35.00
2016	Raji et al	Nigeria	Outpatient	46.30	25.37		Interview	Mini	n/a	205	16.59
2017	Adam et al	USA	Outpatient	28.00	35.40	56.00	PA	BDI-II	14	142	35.21
2017	Dorociak et al	USA	Outpatient	22.20	40.61		PA	CES-D 20	16	54	38.89
2018	Alhomoud et al	Saudi Arabia	Outpatient	57.30	31.70	100	PA	BDI-II	14	110	48.18
2018	Alsubaie et al	Saudi Arabia	Outpatient	35.90	26.40		PA	HAM-D 17	8	78	85.90
2018	Ahmadi et al	Iran	Outpatient	35.10		64.90	PA	DASS 21	5	97	67.01
2018	Williams et al	USA	Inpatient	52.00	27.50		Interview	ED-Scan	n/a	95	29.47
2018	Simo et al	USA	VOC	20.00		80.00	PA	PHQ 9	10	10	50.00

*CES-D* Centre for Epidemiologic Studies-Depression, *BDI* Beck Depression Inventory, *LEMFPSCD* Longitudinal Exploration of Medical and Psychosocial Factors in SCD, *PHQ* Patient Health Questionnaire, *DASS* Depression Anxiety Stress Scale, *BSI* Brief Symptom Inventory, *Mini* Mini International Neuropsychiatric Interview, *PROMIS* Patient-Reported Outcomes Measurement Information System, *HAM-D* Hamilton Depression scale

(^‡^: estimated) (^†^: SS + Sβ°) (*VOC* vaso-occlusive crisis, *PA* psychometric assessment, *MRR* medical record review, *SI* structured interview)

Of the 36 studies considered in this review, 30 used a psychometric assessment (PA) tool: of these, 13 used the Beck Depression Inventory (BDI) [36], 4 used the Centre for Epidemiologic Studies Depression Scale (CES-D) [37], 6 used the Patient Health Questionnaire (PHQ) [38], 2 used the Brief Symptom Inventory (BSI) [39], Depression Anxiety Stress Scale (DASS) [40], 5 used the Longitudinal Exploration of Medical and Psychosocial Factors in SCD (LEMFPSCD), Patient-Reported Outcomes Measurement Information System and Psychosocial Impact of Sickle Cell Disorder (PROMIS, a self-complete questionnaire designed for the study), and 5 used the Hamilton rating scale [41]. A medical record review (MRR) was used in four studies and two studies used a structured interview (SI) (ED-Scan and Mini).

These 36 studies evaluated a total of 6936 adults. In the total sample, 1665 patients were evaluated with a score above the respective cut-off for indicating depression in each of the assessment tools used, indicating that 24.01% of patients have depression. The sample were 41.10% male with a mean age of 32 years, and 79% had the genotype SS.

### Analysis of the methodology used in the included studies

#### Recruitment setting

Most of the studies (n_study_ = 23) used an outpatient population recruited during their normal medical appointment (referred as Outpatient). Some studies used data extracted from a cohort (n_study_ = 7). We grouped under the name cohort studies using either a cohort formed in a longitudinal study framework (e.g. PiSCES) or a subgroup selected from a larger group. In these studies, data were extracted at a single time point. Here, the term cohort study is used to distinguish from general outpatient studies (despite the fact that the data is a snapshot at a single time point and hence transversal) because there is a possibility that the monitoring of patients such studies could affect their perception of their illness (referred as Extracted from a cohort). Five studies explicitly recruited inpatients, and one included patients in vaso-occlusive crisis (VOC), which is a severe painful complication requiring treatment during hospitalization [42] (Referred as Inpatient). Finally, in one study, the sample was constituted with both outpatient and inpatient [43].

Of the 30 studies using a PA tool to assess depression, 21 (70%) recruited outpatients and 7 (23.33%) a cohort. Of the 4 MRR studies, 3 (75%) recruited inpatients and outpatients.

#### Characteristics of the population

The majority of the studies were from the USA (n_study_ = 20), nine from Africa or the Middle East, five from South America and two from France.

Despite one study using a large sample [15], the mean sample size is low (n_patient_ = 193). For studies using psychometric assessment tools, n_patient_ = 138; in medical record review, n_patient_ = 627 but decrease to n_patient_ = 211 when excluding the mentioned study; and, in structured interviews, n_patient_ = 150. Of the recruited patient, 41.10% are male (the breakdown among the different assessment methods is as follows: PA: 40.20%, MRR: 42.80%, SI: 50.00%) with a mean age of 32 years old (PA: 32.70, MRR: 25.60, SI: 30.30) and in the mean at 79% with a genotype SS (PA: 80.10%, MRR: 64%, SI: Not provided).

#### Assessment tool used

The psychometric properties of the instruments used (PA and SI) suggest that these are generally reliably tools, both in their original version (English) and in their adapted versions in other languages. Only one tool was not validated: Psychological Impact of Sickle Cell Disease (PISCD) [44]. The PISCD is a questionnaire designed specifically for use in relation to SCD and has no reported validation. It should also be noted that some articles validating translations of psychometric tools do not report sensitivity and specificity, but provide a measure of internal consistency. This is considered insufficient to guarantee that the psychometric qualities of the original tool are reflected in the translated version. Of course, psychometric tools are screening tools even if they offer discriminating thresholds for levels of intensity of depression.

Specifically, with respect to the Centre for Epidemiologic Studies-Depression, of the four studies using it, three use the 20-item version and a cut-off of 16. However, psychometric studies show that this cut-off does not provide the psychometric qualities required to constitute a valid assessment tool because at this cut-off, specificity is only 61.8% [45]. The Wilson–Schaeffer study does report the prevalence of depression for both a CES-D cut-off of 16 (43.4%), but also for the more stringent 27 (18%). For reasons of consistency between the cut-offs used, we will retain in this study the prevalence provided with the cut-off of 16 (43.4%), while being aware of the low specificity of this cut-off. The fourth study, by Laurence et al., uses the 10-item version which has good psychometric properties.

In the MRR, researchers extracted data of interest from medical records. Of the four MRR, three used diagnostic coding to assess the presence of depression. One [46] used reported symptoms related to depression. These methodologies could have biases that could underestimate depression in that depressed patients who have not yet been diagnosed or whose coding is not recorded will not be included in the research (see Table 2).

**Table 2:** Criteria for depression and possible biases in MRR

Year	Authors	Criteria for depression	Possible biases
2009	Carroll et al	International classification of disease (ICD-9) diagnostic coding for mood disorder (including depressive and bipolar disorders): 296.00 to 296.89	Depressed patients without diagnostic reported Depressed patients diagnosed but code not reported
2011	Carroll et al	ICD-9 diagnostic coding for mood disorder (including depressive and bipolar disorders): 296.00 to 296.89	Depressed patients without diagnostic reported Depressed patients diagnosed but code not reported
2015	Mann-Jiles et al	Reported symptoms related to depression	Other symptoms related to depression were treated separately, for example Anhedonia, Hyper- or hypophagia Insomnia Sleep/wake disturbance A list of symptoms is not enough to hypothesised depression: risk of under- or overestimation
2016	Fayand et al	Diagnostic coding, then Confirmation with medical records by: Mention of opinion, Medical monitoring, Or psychiatric treatment	Depressed patients without diagnostic reported Depressed patients diagnosed but code not reported

### Depression prevalence reported

The prevalence range of depression is very large, from 0% [31] to 85.90% [32]. All studies combined, the mean prevalence of depression is found to be 24.01%. As the sample sizes have a large range, from 10 to 1874, weighted analysis was used. The mean reported prevalence of each study was weighted according to the total size of the sample considered, in order to take into account the contribution of each study when calculating the general and specific mean prevalences.

Table 3 shows the mean of the prevalence reported using the three different categories of instruments employed in the studies. As can be seen from Table 3, prevalence appears to depend on the type of assessment tool employed. MRR is associated with strikingly lower prevalence (close to 5%) than PA or SI (which are close to 36% and 21% respectively).

**Table 3:** Prevalence of depression according to assessment instruments used

Tool	n_patients_	Mean (%)	Minimum (%)	Maximum (%)
PA (n_study_ = 30)	4129	35.80	00.00	85.90
MRR (n_study_ = 4)	2507	04.99	04.27	19.40
SI (n_study_ = 2)	300	20.67	16.60	29.50

*PA* psychometric assessment, *MRR* medical records reviews, *SI* structured interview

#### Effect of the geographic region

To examine any possible regional influence on the prevalence of depression, we have grouped results according to the location of the studies. The regional regrouping were the USA, South America, Europe, and, Africa-Middle East. Because of the apparent influence of the measurement instrument, only studies that used PA tools were considered (Table 4) when comparing the prevalence in different regions. The results suggest that South America has the lowest prevalence of depression at almost 20%, and that Africa/Middle East has the highest at almost 47%. The USA has an intermediate prevalence at about 34%. The single study from Europe indicated a relatively high prevalence of close to 49%.

**Table 4:** Depression mean prevalence according to the continent, time, and setting, considering only assessment with psychometric tools

	n_patients_	Mean (%)	Minimum (%)	Maximum (%)
*Continent*				
The USA (n_study_ = 16)	2138	34.33	20.63	61.04
South America (n_study_ = 5)	694	19.74	13.29	30.00
Europe (n_study_ = 1)	89	49.44	–	–
Africa/Middle East (n_study_ = 8)	1208	46.61	0.00	71.84
*Time*				
1999–2000 (n_study_ = 2)	483	41.82	25.58	43.41
2001–2010 (n_study_ = 10)	1653	36.30	21.66	59.26
2011–2020 (n_study_ = 18)	1993	33.92	0.00	71.84
*Setting*				
Inpatient and VOC (n_study_ = 1)	10	50.00	–	–
Outpatient (n_study_ = 21)	2706	39.84	0.00	71.84
Extracted from a cohort (n_study_ = 7)	1290	26.90	20.63	38.61

*VOC* vaso-occlusive crisis

#### Effect of time of measurement on the level of depression measured

We have divided the results into 10-year intervals, again only considering studies employing PA tools (Table 4). Two observations can be made: the number of studies and number of participants increase with time, and the prevalence of depression appears to decrease with time.

#### Effect of setting of recruitment

We have divided the data according to the setting of recruitment: inpatient and VOC, outpatient, and cohorts (Table 4), considering only studies using PA tools. It appears that the outpatient prevalence is nearly 13 points higher than the cohort prevalence. However, scarcity of data available in the Inpatient group (n_study_ = 1) is a limit for further comparison.

### Data synthesis: depression in awSCD in the USA

The USA provides by far the largest number of studies included in this review. Of the 20 studies considered, 16 used a psychometric assessment of depression, 3 used a medical record review, and 1 used a structured interview. These studies evaluated 4301 adults with a mean age of 33.21 years. Of these, 39.72% were male (the breakdown among the different assessment methods is as follows: PA: 38.3%, MRR: 42.80%, SI: 52.00%) and 73.91% SS (PA: 75.6%, MRR: 64%, SI: -).

In the USA, the mean prevalence of depression is calculated to be 20.09% (min/max [4.27%; 61%]), using data from all the studies. Differences appear according to the evaluation tool used (PA: 34.33%, MRR: 4.93%, SI: 29.47%) (Table 5). MRR appears to have the smallest mean prevalence level which is consistent with our previous findings. In view of the lack of reliability of the MRRs, they were excluded from analysis according to time and to recruitment setting (Table 5).

**Table 5:** Mean depression prevalence according to tools used, time of measurement, and recruitment setting for the United State

	n_patients_	Mean (%)	Minimum (%)	Maximum (%)
*Tool*				
PA (n_study_ = 16)	2138	34.33	20.63	61.04
MRR (n_study_ = 3)	2068	4.93	4.27	19.44
SI (n_study_ = 1)	95	29.47	–	–
*Time (excluding MRR)*				
1999–2000 (n_study_ = 2)	483	41.82	25.58	43.41
2001–2010 (n_study_ = 7)	880	32.27	22.39	44.00
2011–2020 (n_study_ = 8)	870	31.72	20.63	61.04
*Setting (excluding MRR)*				
Inpatient and VOC (n_study_ = 1)	10	50	–	–
Outpatient (n_study_ = 9)	1115	39.62	22.39	61.04
Extracted from a cohort (n_study_ = 6)	1013	28.33	20.63	38.61

*PA* psychometric assessment, *MRR* medical records reviews, *SI* structured interview, *VOC* vaso-occlusive crisis

In a decade by decade perspective, if we exclude the two studies for the period 1999–2000, the prevalence of depression is stable around 32.00% (99–2000: 41.82%, 2001–10: 32.27%, 11–20: 31.72%).

Depending on the recruitment setting, prevalence appears to be different. In inpatient and VOC groups, prevalence is close to 6%, almost 40% in outpatient group, and 28% in the cohort. This result is equivalent to what we found previously.

The results keeping the regional variable and assessment tool constant are consistent with those previously presented, confirming that MRR tends to provide a lower estimate of the prevalence of depression than PA or SI, questioning us on its use in studies.

## Discussion

This literature review has revealed several key points. First, there is a very large difference in the prevalence levels that have been reported, and there are wide differences in prevalence observed across different regions. The mean of all the studies indicates a prevalence of 24%, but the range extends from 0% [31] to more than 85% [32]. The extremities of the range appear to be due to methodological issues. On the one hand, Anim calculates prevalence by dividing the “*mean number of individuals in the SCD sample who had indicated non-zero responses*” by the total population included [31]. However, prevalence is calculated by dividing the number of cases over the population as the authors specify in their methodology (idem, p. 4). By using an average of the cases, Anim (2016) artificially reduce the reported prevalence. On the other hand, with the Hamilton Depression Rating Scale, Alsubaie (2018) calculate prevalence of depression by assuming that all the score above or equal to 8 indicates a depression regardless of the severity of the depression. However, empirical research has established that this cut-off is too low, and that raised cut-off should be employed [47]. In fact, a cut-off as high as 17 has been proposed [48] to discriminate between depressed and non-depressed patients.

Second, the different measurement tools used in themselves seem to provide very different results. Medical record reviews suggest a prevalence of 5%, whereas psychometric assessment tools tend to indicate a much higher prevalence (36%), as do structured interviews (21%).

In the four articles using a medical record review, two methodologies are used to define the cases. Three articles use diagnostic codes, the last article from Mann-Jiles uses symptoms related to depression. The relatively low prevalence reported with medical record-based assessments is thought to an underestimate due to two methodological biases. First, authors using the diagnostic codes included all the patients with a diagnosis coded between ICD 9 F-296.00 and F-296-89 corresponding, not only to the major depressive disorders, but also to the entire spectrum of bipolar disorders. We would expect to obtain a significant prevalence, which is not the case. These three articles provide the lowest prevalence of depression. One explanation for this low prevalence could be the fact that diagnostic codes of depression are known to be under-reported in administrative records [49]. Then, with regard to the Mann–Jiles article specifically, the authors relied on the presence of certain symptoms relating to depression to label the patient as depressed. However, the authors decided to treat separately some symptoms directly linked to depression such as anhedonia, hyper or hypophagy, insomnia and sleep disorders. This methodological choice, consisting in a particularly restricted definition of depression, may contribute to the weaker recognition of the number of depressed patients, which is not the case. A prevalence close to 20% is found. Overall, there is a contradiction between the methodologies used and the results found. As a consequence, since it is impossible for researchers to estimate the prevalence of underreporting of the diagnosis of depression in medical records, it should be recommended not to use MRRs as a means of estimation of the prevalence of depression.

In respect of psychometric assessment, little attention has been paid to the cross-cultural context in which assessments occurred. A discrepancy can arise between the cultural context in which the assessment tool has been created and the one in which it is used [50]. In that sense, validations of psychometric tools that are only based on translation and internal consistency fail to prove that what they are aimed to measure is really measured. Research has proven that expression of depression varies between culture [51]. In a western context, emotional symptoms are preponderant to somatic symptoms, but this balance might be reversed in other cultures [52]. This issue should be kept in mind when assessing depression in another context than western countries.

Moreover, as with many other chronic conditions, obtaining an accurate diagnosis of depression in sickle cell disease is challenging [53]. Challenges include overlap of neurovegetative symptoms (fatigue and fatiguability, sleep disturbance, and physical pain) found in both depression and sickle cell disease. This challenge is particularly relevant in psychometric assessment completed during hospitalisation when the intensity of symptoms is increased, especially pain and fatigue. Consequently, prevalence of depression found in the five studies that explicitly include inpatients are questionable. Furthermore, due to the episodic nature of depression, a cross-sectional methodology or a single assessment (e.g. 54), can lead to missed cases. It is pointed out that Hospital Anxiety and Depression Scale (HADS) [54] avoids neurovegetative symptoms and hence would be particularly suitable for use in with chronically ill patients in a hospital setting.

To take into account any possible geographical effect on prevalence level, we examined more closely studies from the United States (n_patient_ = 20, 56%). Here, we find again that psychometric assessment indicates a slightly higher prevalence (34%) than structured interviews (29%). Given the relatively large number (n_study_ = 16) of studies using psychometric assessment, and the total number of participants in these assessments (n_patient_ = 2138), we tentatively suggest that the true extent of prevalence in the USA may be close 34%.

One interesting finding is that the prevalence appears to have decreased globally over the last 20 years when considering studies using only psychometric assessment (Table 4). This may be because the quality of life with the disease has improved or because of the progress in the global management of sickle cell disease and the introduction of new treatments in the last 20 years [55, 56]. However, in the USA this decrease appears to plateau. This is interesting given that since the first introduction of hydroxyurea in 1985 [57] in patients with sickle cell disease, in the United States, and the demonstration of its clinical efficacy in 1995 [58], the quality of care has continued to increase in the United States. Thus, the stagnation of the level of prevalence of depression found in the USA suggests that the improvement of the psychological well-being of the patients cannot be reduced to a therapeutic improvement and that other psychological factors are at play in patients suffering from chronic disease.

From a more general point of view, the socio-demographic or medical characterisation of the samples is incomplete (Table 1). Almost 17% (n = 6) of the studies did not indicate a sex ratio for their overall sample, more than 22% (n = 8) of the studies did not provide clear indications on the age of the patients and 50% ( n = 18) of them do not give the genotypic composition of the patients included. The absence of this information contributes to the poor methodological quality found in the studies.

## Conclusion

Studies on the prevalence of depression in pwSCD over a twenty-year period have been examined. The reported prevalence level of depression of pwSCD appears to vary widely depending on the study in question, and in particular on the assessment tool employed. Given that semi-structured interviews are considered the gold standard for diagnostic assessment, it is surprising that only two studies used it, even if cost and time often limit their use. The result of the study using this technique provides a prevalence of 29%, a level that is relatively close to that provided by psychometric assessment (36%) across 30 studies, we tentatively conclude that the true level of prevalence is somewhere between these two figures, rather than the much lower levels of around 5% reported using medical record reviews.

We note that studies across different geographical regions provide different results, but the small number of studies and methodological issues arising in some of these studies precludes us from making drawing any firm conclusions from this. However, an analysis of studies only from the US did not put in question our general findings outlined above. One interesting finding, again only considering studies from the US, was that the prevalence of depression appears to be stagnant with time, which may be due to the improved treatment options for pwSCD and the fact that improving mental well-being cannot be reduced to therapeutic improvement. Clearly, more needs to be done to improve access to treatment, and it is hoped that the analysis of depression in pwSCD reported here will be a useful contribution to understanding how to tackle this issue.

In the future, it would be useful to conduct studies using both structured interviews and psychometric assessment in order to calibrate the psychometric tools employed with pwSCD, and to better understand the discrepancies found between the two techniques. In particular, regarding the limitations of other psychometric tools, the use of HADS is recommended to avoid overlap between symptoms of depression and those of sickle cell disease. It would also be helpful for more studies to be conducted in regions other than the USA and Europe in order to understand the impact of different healthcare systems and cultures on the prevalence of depression with pwSCD.

## Data Availability

The dataset generated and analysed during this study are included in this published article (Table 1).

## References included in the review (alphabetical order)

- Adam, S. S., Flahiff, C. M., Kamble, S., Telen, M. J., Reed, S. D., & De Castro, L. M. (2017). Depression, quality of life, and medical resource utilization in sickle cell disease. Blood Advances, 1(23), 1983–1992.
- Ahmadi, M., Poormansouri, S., Beiranvand, S., & Sedighie, L. (2018). Predictors and Correlates of Fatigue in Sickle Cell Disease Patients. International Journal of Hematology-Oncology and Stem Cell Research, 12(1), 69–76.
- Al Sherawi, M., Al Alawi, Al Alawi, M., Al Sinawi, H., & Al Farsi, K. (2016). Depressive Symptoms among Patients in a Clinic for Sickle Cell Disease in Oman: A Cross—Sectional Study. The Arab Journal of Psychiatry, 27(1), 59–66. https://doi.org/10.12816/0023157
- Alhomoud, M. A., Gosadi, I. M., & Wahbi, H. A. (2018). Depression among Sickle Cell Anemia Patients in the Eastern Province of Saudi Arabia. Saudi Journal of Medicine & Medical Sciences, 6(1), 8–12. https://doi.org/10.4103/sjmms.sjmms_123_16
- Alsubaie, S. S., Almathami, M. A., Abouelyazid, A., & Alqahtani, M. M. (2018). Prevalence of depression among adults with sickle cell disease in the southern region of Saudi Arabia. Pakistan Journal of Medical Sciences, 34(4), 929–933. https://doi.org/10.12669/pjms.344.14760
- Anie, K. A., Egunjobi, F. E., & Akinyanju, O. O. (2010). Psychosocial impact of sickle cell disorder: Perspectives from a Nigerian setting. Globalization and Health, 6(1), 2.
- Anim, M. T., Osafo, J., & Yirdong, F. (2016). Prevalence of psychological symptoms among adults with sickle cell disease in Korle-Bu Teaching Hospital, Ghana. BMC Psychology, 4(1). https://doi.org/10.1186/s40359-016-0162-z
- Asnani, M. R., Fraser, R., Lewis, N. A., & Reid, M. E. (2010). Depression and loneliness in Jamaicans with sickle cell disease. BMC Psychiatry, 10(1), 40.
- Carroll, C. P., Haywood, C., Fagan, P., & Lanzkron, S. (2009). The course and correlates of high hospital utilization in sickle cell disease: Evidence from a large, urban Medicaid managed care organization. American Journal of Hematology, 84(10), 666–670. https://doi.org/10.1002/ajh.21515
- Carroll, C. P., Haywood, C., & Lanzkron, S. (2011). Prediction of onset and course of high hospital utilization in sickle cell disease. Journal of Hospital Medicine, 6(5), 248–255. https://doi.org/10.1002/jhm.850
- Dorociak, K. E., Schulze, E. T., Piper, L. E., Molokie, R. E., & Janecek, J. K. (2018). Performance validity testing in a clinical sample of adults with sickle cell disease. The Clinical Neuropsychologist, 32(1), 81–97. https://doi.org/10.1080/13854046.2017.1339830
- Edwards, C., Green, M., Wellington, C., Muhammad, M., Wood, M., Feliu, M., Edwards, L., Sollers, J. 3rd, Barksdale, C., Robinson, E., McDougald, C., Abrams, M., Whitfield, K., Byrd, G., Hubbard, B., Cola, M., De Castro, L. M., & McNeil, J. (2009). Depression, suicidal ideation, and attempts in black patients with sickle cell disease. Journal of the National Medical Association, 101(11), 1090–1095.
- Fayand, A., Dzierzynski, N., Georgin-Lavialle, S., Lionnet, F., & Steichen, O. (2016). Influence des comorbidités psychiatriques sur le recours hospitalier pour crise vaso-occlusive chez l’adulte drépanocytaire: Une étude de cohorte. La Revue de Médecine Interne, 37, A67–A68. https://doi.org/10.1016/j.revmed.2016.10.017
- Gibson, R. C., Morgan, K. A. D., Abel, W. D., Sewell, C. A., Martin, J. S., Lowe, G. A., Haye, W. D. L., Edwards, C. L., O’Garo, K. N., Reid, M. E., & Asnani, M. R. (2013). Locus of control, depression and quality of life among persons with sickle cell disease in Jamaica. Psychology, Health & Medicine, 18(4), 451–460. https://doi.org/10.1080/13548506.2012.749353
- Grant, M. M., Gil, K. M., Floyd, M. Y., & Abrams, M. (2000). Depression and functioning in relation to healthcare use in sickle cell disease. Annals of Behavioral Medicine, 22(2), 149–157.
- Hasan, S. P., Hashmi, S., Alhassen, M., Lawson, W., & Castro, O. (2003). Depression in sickle cell disease. Journal of the National Medical Association, 95(7), 533–537.
- Jenerette, C., Funk, M., & Murdaugh, C. (2005). Sickle cell disease: A stigmatizing condition that may lead to depression. Issues in Mental Health Nursing, 26(10), 1081–1101. https://doi.org/10.1080/01612840500280745
- Kamble, S., Reed, S. D., Flahiff, C., Adam, S., & DeCastro, L. M. (2010). Resource Use and Expenditures Among Adult Sickle Cell Patients with and without Depression. Blood, 116(21), 1534.
- Laurence, B., George, D., & Woods, D. (2006). Association between elevated depressive symptoms and clinical disease severity in African-American adults with sickle cell disease. Journal of the National Medical Association, 98(3), 365.
- Levenson, J. L., McClish, D. K., Dahman, B. A., Bovbjerg, V. E., de A. Citero, V., Penberthy, L. T., Aisiku, I. P., Roberts, J. D., Roseff, S. D., & Smith, W. R. (2008). Depression and Anxiety in Adults With Sickle Cell Disease: The PiSCES Project: Psychosomatic Medicine, 70(2), 192–196. https://doi.org/10.1097/PSY.0b013e31815ff5c5
- Mahdi, N., Al-Ola, K., Khalek, N. A., & Almawi, W. Y. (2010). Depression, anxiety, and stress comorbidities in sickle cell anemia patients with vaso-occlusive crisis. Journal of Pediatric Hematology/Oncology, 32(5), 345–349.
- Mann-Jiles, V., Thompson, K., & Lester, J. (2015). Sleep impairment and insomnia in sickle cell disease: A retrospective chart review of clinical and psychological indicators: Sleep impairment and insomnia in sickle cell disease. Journal of the American Association of Nurse Practitioners, 27(8), 441–449. https://doi.org/10.1002/2327-6924.12212
- Mastandréa, É. B., Lucchesi, F., Kitayama, M. M. G., Figueiredo, M. S., & Citero, V. de A. (2015). The relationship between genotype, psychiatric symptoms and quality of life in adult patients with sickle cell disease in São Paulo, Brazil: A cross-sectional study. São Paulo Medical Journal, 133(5), 421–427. https://doi.org/10.1590/1516-3180.2015.00171105
- Master, S., Arnold, C., Davis, T., Shi, R., & Mansour, R. P. (2016). Anxiety, Depression, Pain Intensity and Interference in Adult Patients with Sickle Cell Disease. Blood, 128(22), 1312–1312.
- Morgan, K. A. D., Scott, J.-K., Parshad-Asnani, M., Gibson, R. C., O’Garo, K. N., Lowe, G. A., Edwards, D., Abel, W. D., Reid, M., De La Haye, W., & Edwards, C. L. (2014). Associations among disease severity, religious coping and depression in a cohort of Jamaicans with sickle-cell disease. Mental Health, Religion & Culture, 17(9), 937–945. psyh. https://doi.org/10.1080/13674676.2014.961910
- Ola, B. A., Yates, S. J., & Dyson, S. M. (2016). Living with sickle cell disease and depression in Lagos, Nigeria: A mixed methods study. Social Science & Medicine, 161, 27–36. https://doi.org/10.1016/j.socscimed.2016.05.029
- Pells, J., Edwards, C. L., McDougald, C. S., Wood, M., Barksdale, C., Jonassaint, J., Leach-Beale, B., Byrd, G., Mathis, M., & Harrison, M. O. (2007). Fear of movement (kinesiophobia), pain, and psychopathology in patients with sickle cell disease. The Clinical Journal of Pain, 23(8), 707–713.
- Raji, S. O., Lawani, A. O., & James, B. O. (2016). Prevalence and correlates of major depression among Nigerian adults with sickle cell disease. The International Journal of Psychiatry in Medicine, 51(5), 456–466. https://doi.org/10.1177/0091217416680839
- Rosine, M. (2012). Répression émotionnelle, dépression et stratégie de ‘coping’ face à la douleur chez l’adulte souffrant de drépanocytose [Etude observationnelle de 89 sujets]. Université de Lorraine.
- Simo, S. M., & Siela, D. (2018). Use of a depression and sleep impairment treatment guideline to improve quality of life for patients with sickle cell disease. International Journal of Palliative Nursing, 24(5), 246–255. https://doi.org/10.12968/ijpn.2018.24.5.246
- Treadwell, M. J., Barreda, F., & Kaur, K. (2015). Emotional Distress, Barriers to Care, and Health-Related Quality of Life in Sickle Cell Disease. Journal of Clinical Outcomes Management, 22(1), 10–20.
- Treadwell, M. J., Barreda, F., Major, K., Walker, V., Payton, W., Kaur, K., & Vichinsky, E. (2011). Mental Health Symptoms, Quality of Life and Barriers to Accessing Health Care in Sickle Cell Disease. Blood, 118(21), 337–337.
- Vilela, R. Q. B., Cavalcante, J. C., Cavalcante, B. F., Araújo, D. L., Lôbo, M. de M., & Nunes, F. A. T. (2012). Quality of life of individuals with sickle cell disease followed at referral centers in Alagoas, Brazil. Revista Brasileira de Hematologia e Hemoterapia, 34(6), 442–446. https://doi.org/10.5581/1516-8484.20120110
- Wallen, G. R., Minniti, C. P., Krumlauf, M., Eckes, E., Allen, D., Oguhebe, A., Seamon, C., Darbari, D. S., Hildesheim, M., Yang, L., & others. (2014). Sleep disturbance, depression and pain in adults with sickle cell disease. BMC Psychiatry, 14(1), 1.
- Williams, H., Silva, S., Cline, D., Freiermuth, C., & Tanabe, P. (2018). Social and Behavioral Factors in Sickle Cell Disease: Employment Predicts Decreased Health Care Utilization. Journal of Health Care for the Poor and Underserved, 29(2), 814–829. https://doi.org/10.1353/hpu.2018.0060
- Wilson Schaeffer, J. J., Gil, K. M., Burchinal, M., Kramer, K. D., Nash, K. B., Orringer, E., & Strayhorn, D. (1999). Depression, disease severity, and sickle cell disease. Journal of Behavioral Medicine, 22(2), 115–126.

## Abbreviations

awSCD: Adults with sickle cell disease
BDI: Beck Depression Inventory
BSI: Brief Symptom Inventory
CES-D: Centre for Epidemiologic Studies Depression Scale
DASS: Depression Anxiety Stress Scale
HADS: Hospital Anxiety and Depression Scale
HAM-D: Hamilton Depression Scale
ICD-9: International classification of diseases 9^th^ edition
LEMFPSCD: Longitudinal Exploration of Medical and Psychosocial Factors in SCD
MRR: Medical record review
PHQ: Patient Health Questionnaire
PISCD: Psychological Impact of Sickle Cell Disease
PRISMA: Preferred Reporting Items for Systematic Reviews and Meta-Analyses
PROMIS: Patient-Reported Outcomes Measurement Information System and Psychosocial Impact of Sickle Cell Disorder
pwSCD: People with sickle cell disease
SCD: Sickle cell disease
SI: Structured interview
VOC: Vaso-occlusive crisis
